# A mathematical model for three-phase-lag dipolar thermoelastic bodies

**DOI:** 10.1186/s13660-017-1380-5

**Published:** 2017-05-10

**Authors:** M Marin, RP Agarwal, L Codarcea

**Affiliations:** 1 Department of Mathematics and Computer Science, Transilvania University of Brasov, Brasov, 500091 Romania; 2 Department of Mathematics, Texas A&M University-Kingsville, Kingsville, TX 78363 USA

**Keywords:** dipolar bodies, three-phase-lag, reciprocal relation, uniqueness result, variational principle equation

## Abstract

In this study we approach a mixed initial-boundary value problem to modeling a three-phase-lag dipolar thermoelastic body. The constitutive laws in this context are given. We establish a uniqueness result and prove a reciprocal theorem. The variational principle obtained in this context is a generalization of the known Gurtin’s principle for classical elasticity.

## Introduction

In many studies published in the last period of time it was demonstrated that the classical uncoupled theory of thermoelasticity predicts two phenomena not compatible with concrete experiments: the equation of heat conduction does not contain any elastic terms and the heat equation is of parabolic type and this means that it predicts infinite speeds of propagation for heat waves. As such there are published a number of studies to eliminate the paradoxes of the classical theory. We refer to some of them, most of them are discussed lately. The first important model is due to Lord and Shulman [1]. Then Green and Naghdi developed three different theories, labeled type I, type II and type III, in [2–4]. We want to outline that the Green-Naghdi theory of type I is equivalent to the classical coupled thermoelasticity theory; the Green-Naghdi theory of type II does not admit energy dissipation and implies a finite speed of propagation for heat waves; and the Green-Naghdi theory of type III admits dissipation of energy and the heat flux is a combination of type I and type II. Also, this type III implies a finite speed of propagation for heat waves. In other recent studies (see, for instance, [5]), the Fourier law is replaced by an approximation of the equation where the thermal displacement function, the thermal conductivity tensor and the conductivity rate tensor appear. This is the three-phase-lag heat conduction model, and it is an extension of the dual-phase-lag (see [6]). Using Taylor approximations, it is proved that this theory covers the Green-Naghdi theories. Many studies that highlight the beneficial effect in terms of application of this theory have been published. So, the effects of three-phase-lags on an infinite medium with cylindrical cavity are studied in [7], and in [8] the generalized thermoelastic functionally graded orthotropic hollow sphere under thermal shock with three-phase-lag effect is studied. Other studies emphasize the theoretical advantages of the three-phase-lag theory. So, in [9] and [10] the authors highlight the importance of the reciprocity theorems which produce boundary integral equations used in the boundary element method. Also, the variational principles provide a theoretical basis for numerical techniques such as finite-element method or boundary element method (see [11–14]).

The theory of bodies with microstructure has the primary aim to remove the differences which occur between experiments and the classical theory of elasticity. The results of classical elasticity prove not to be appropriate when the body’s overall deformations are subject to effects of material microstructure. This happens in the case of ceramics, graphite, human bones, polymers (that is, some granular bodies with large molecules), and so on. Eringen was first to study this kind of theory (see, for instance, [15, 16]) which was continuously studied in various papers, such as [17–20]. Some considerations on waves for specific bodies with microstructure can be found in [21–24]. A specific aspect of the microstructure is the dipolar structure. Many valuable researchers emphasized the importance of the dipolar structure of materials. The start was given by the published results of Mindlin [25] as well as Green and Rivlin [26], who approached also in other papers the multipolar structures and, in particular, the dipolar structures. Another known researcher, Gurtin, has published a few articles on multipolar structures. It is enough to recall the paper [27] where Gurtin together with Fried discover integral statements of force balance, energy balance and entropy imbalance for an interface between a body and its environment. We want to outline that in the theory of dipolar continua the degrees of freedom for each particle are three translations and nine micro-deformations, and each material point is constrained to deform homogeneously. The theories of dipolar bodies are quite sufficient for a large number of applications in solid mechanics.

In our present study the constitutive equations for the three-phase-lag dipolar thermoelastic solids are given. These were obtained by the procedure used in a simple case of classical elasticity. In order to prove the consistency of the mixed problem formulated in this context, we state and argue two qualitative results, namely uniqueness and reciprocal results. These are obtained without resorting to known processes (for example, without using Laplace’s transform). Instead of these procedures, in proving the uniqueness result, the dissipative inequality is used. Also, for the general case of an anisotropic and inhomogeneous material, we prove a variational principle which generalizes the known convolutional variational principle of Gurtin in order to cover the three-phase-lag dipolar thermoelasticity theory.

## Notations and basic equations

Assume that at time $t_{0}$ our dipolar thermoelastic body occupies the domain Ω included in the Euclidean three-dimensional space $R^{3}$. Its boundary is the piecewise smooth surface *∂*Ω. In Ω we will use a fixed system of rectangular Cartesian axes $Ox_{i}, i=1,2,3$, so that in this system any point *P* from Ω is characterized by three rectangular coordinates $x_{1}, x_{2}, x_{3}$, and we use the notation *x* for $( x_{1},x _{2},x_{3} ) $. So, *x* will be the position and *t* will be time. The functions in the following are considered to be functions of $(x,t)$ defined on cylinder $\bar{\Omega }\times (0,\infty )$, where $\bar{\Omega }=\Omega \cup \partial \Omega $. If there is no likelihood of confusion, the spatial variables and the time variable of functions will be omitted. We will use the known convention on summation over repeated subscripts and differentiation. Greek subscripts are understood to range over the integers $(1,2)$, and Latin subscripts take the values $1,2,3$. We also use a superposed dot to denote the partial differentiation with respect to time, *t*, $\dot{f}=\partial f/\partial t$, and a subscript preceded by a comma denotes partial differentiation with respect to the corresponding Cartesian coordinate, $f_{,j}= \partial f/\partial x_{j}$. The motion of the body will be characterized by the displacement vector of components $( u_{i} ) $, the dipolar displacement tensor of components $( \varphi_{ij} ) $ and variation of temperature *θ*. We will consider that all components of displacement and temperature variation from some reference temperature are small. Also, the space derivatives of these functions and their time derivatives are small.

Our mathematical model requires a system of governing equations in the context of the linear theory of dipolar thermoelasticity. Using the known procedure of Green and Rivlin, we consider a new motion which differs from the given motion only by superposed rigid motion defined by a rotation of uniform rigid body angular velocity; and suppose that for the given motion, all characteristics of the body are unaltered by such superposed rigid motion. So we deduce the following kinetic relations, which give expressions of the strain measures $\varepsilon _{ij}$, $\gamma_{ij}$ and $\chi_{ijk}$ with regard to variables of motion (see Eringen [15]):

1 $$\begin{aligned} \varepsilon_{ij}=\frac{1}{2} ( u_{i,j}+u_{j,i} ) ,\qquad \gamma _{ij}=u_{j,i}- \varphi_{ij}, \qquad \chi_{ijk}=\varphi_{jk,i}. \end{aligned}$$

Also, according to Eringen [15], the continuity equation has the form

2 $$\begin{aligned} \frac{d\varrho }{dt}+\varrho \dot{u}_{j,j}=0, \end{aligned}$$

where *ϱ* is the mass density.

With a suggestion given by Green and Naghdi in [2], we will consider the thermal displacement *α* and thermal gradient $\beta_{i}$ defined with the help of temperature by

3 $$\begin{aligned} \alpha (x,t)= \int_{t_{0}}^{t} \theta (x,\tau )\,d\tau ,\qquad \beta_{i} (x,t)= \int_{t_{0}}^{t} \theta_{,i}(x,\tau )\,d\tau . \end{aligned}$$

Clearly, we have

$$ \dot{\alpha }(x,t)=\theta (x,t),\qquad \alpha (x,t_{0})=0,\qquad \beta_{i} (x,t)= \alpha_{,i} (x,t), $$

and, in the case $t_{0}=0$, we obtain

4 $$\begin{aligned} \alpha (x,0)=0,\qquad \beta_{i} (x,0)=0. \end{aligned}$$

Now we obtain some consequences of the laws of thermodynamics. So it is required that the first two laws of thermodynamics hold at every time *t* and at any point $x\in \Omega $.

If we denote by $n_{i}$ the components of the outward unit normal to the surface *∂*Ω, then at each regular point of *∂*Ω we can define the components of surface traction $t_{i}$, the components of surface couple $\mu_{jk}$ and the surface heat flux *q* by

5 $$\begin{aligned} t_{i}= ( \tau_{ij}+\sigma_{ij} ) n_{j}, \qquad m_{jk}=\mu_{ijk}n _{i},\qquad q=q_{i}n_{i}, \end{aligned}$$

where $\tau_{ij}$, $\sigma_{ij}$ and $\mu_{ijk}$ are the components of the stress and $q_{i}$ are the components of the heat flux vector.

Now, we denote by *e* the internal energy per unit mass, by $I_{ij}$ the micro-inertia tensor (which is a symmetric tensor), by $f_{i}$ the components of body force, by $g_{jk}$ the components of body couple and by *Q* the intensity of applied heat source per unit volume. Then the first law of thermodynamics is

6 $$\begin{aligned}& \frac{d}{dt} \int_{\Omega } ( \varrho e+\varrho \dot{u}_{i}\dot{u} _{i}+I_{jk}\dot{\varphi }_{js}\dot{\varphi }_{ks} )\,dV \\& \quad = \int_{\Omega } ( \varrho f_{i}\dot{u}_{i}+ \varrho g_{jk} \dot{\varphi }_{jk}+Q )\,dV+ \int_{\partial \Omega } ( t_{i} \dot{u}_{i}+m_{jk} \dot{\varphi }_{jk}-q )\,dA. \end{aligned}$$

Also, if *η* is the entropy per unit volume and *T* is the absolute temperature, then the second law of thermodynamics receives the form

7 $$\begin{aligned} \dot{\eta }\ge \frac{Q}{T}-\frac{q_{i,i}}{T}+ \frac{q_{i}}{T^{2}}T_{,i},\quad T=T_{0}+\theta , \end{aligned}$$

where $T_{0}$ is the temperature in the reference state.

With the help of equations (1) and (2) and using the divergence theorem, from (6) we deduce

8 $$\begin{aligned}& \varrho \dot{e}=\tau_{ij}\dot{\varepsilon }_{ij}+\sigma_{ij} \dot{\gamma }_{ij}+ \mu_{ijk}\dot{\chi }_{ijk}+Q-q_{i,i}, \\& ( \tau_{ij}+\sigma_{ij} ) _{,j}+\varrho f_{i}=\varrho \ddot{u}_{i}, \\& \mu_{ijk,i}+\sigma_{jk}+\varrho g_{jk}=I_{js} \ddot{\varphi }_{ks}. \end{aligned}$$

Considering (8)_1_, inequality (7) becomes

9 $$\begin{aligned} \varrho \dot{e}-\tau_{ij}\dot{\varepsilon }_{ij}-\sigma_{ij} \dot{\gamma }_{ij}- \mu_{ijk}\dot{\chi }_{ijk}-T\dot{\eta }+ \frac{q _{i} T_{, i}}{T}\le 0. \end{aligned}$$

Now we introduce the Helmholtz free energy density, denoted by Ψ, and defined by

10 $$\begin{aligned} \Psi =\varrho e-T\eta, \end{aligned}$$

such that the entropy inequality (9) receives the form

11 $$\begin{aligned} \dot{\Psi }+\dot{T}\eta -\tau_{ij}\dot{\varepsilon }_{ij}-\sigma_{ij} \dot{\gamma }_{ij}- \mu_{ijk}\dot{\chi }_{ijk}+ \frac{q_{i} \theta_{, i}}{T}\le 0. \end{aligned}$$

Taking into account that the set of independent variables of function Ψ is $( \varepsilon_{ij},\gamma_{ij}, \chi_{ijk},\theta ) $, from (11) we are led to

12 $$\begin{aligned} \frac{\partial \Psi }{\partial \varepsilon_{ij}}\dot{\varepsilon } _{ij} + \frac{\partial \Psi }{\partial \gamma_{ij}}\dot{\gamma } _{ij} + \frac{\partial \Psi }{\partial \chi_{ijk}}\dot{\chi }_{ijk} + \frac{\partial \Psi }{\partial \theta }\dot{\theta } + \dot{\theta }\eta - \tau_{ij}\dot{\varepsilon }_{ij} - \sigma_{ij}\dot{ \gamma }_{ij} - \mu_{ijk}\dot{\chi }_{ijk} + \frac{q _{i} \theta_{, i}}{T} \le 0. \end{aligned}$$

We restrict our considerations to the case where the materials have a center of symmetry. Also, we suppose that the body is free from stress, in its reference configuration, and has zero intrinsic equilibrated body forces and body couples. The linear theory requires a quadratic form for the internal energy density with regard to its independent constitutive variables. According to the principle of conservation of energy, we can expand in series the internal energy density about reference configuration so that it can be written in the following form:

13 $$\begin{aligned} \Psi& = \frac{1}{2}C_{ijmn}\varepsilon_{ij} \varepsilon_{mn}+ G_{ijmn} \varepsilon_{ij} \gamma_{mn}+ F_{ijmnr}\varepsilon_{ij} \chi_{mnr}+ \frac{1}{2}B_{ijmn}\gamma_{ij} \gamma_{mn} \\ &\quad {}+D_{ijmnr}\gamma_{ij}\chi_{mnr} +\frac{1}{2}A_{ijkmnr} \chi_{ijk}\chi _{mnr}- {\tt a}_{ij} \varepsilon_{ij}\theta - b_{ij}\gamma_{ij}\theta - c_{ijk}\chi_{ijk}\theta - \frac{1}{2}\beta \theta^{2}. \end{aligned}$$

As a consequence, this form of Ψ is used in inequality (12) to obtain the constitutive equations that give the expressions for stress measures in terms of the strain measures

14 $$\begin{aligned}& \tau_{ij}= \frac{\partial \Psi }{\partial \varepsilon_{ij}}= C_{ijmn} \varepsilon_{mn}+G_{mnij} \gamma_{mn}+F_{mnrij} \chi_{mnr}- {\tt a} _{ij}\theta , \\& \sigma_{ij}= \frac{\partial \Psi }{\partial \gamma_{ij}}= G_{ijmn} \varepsilon_{mn}+B_{ijmn} \gamma_{mn}+D_{ijmnr} \chi_{mnr}- b_{ij} \theta , \\& \mu_{ijk} = \frac{\partial \Psi }{\partial \chi_{ijk}}= F_{ijkmn} \varepsilon_{mn} + D_{mnijk} \gamma_{mn} + A_{ijkmnr} \chi_{mnr} - c_{ijk}\theta , \\& \eta = -\frac{\partial \Psi }{\partial \theta }= {\tt a}_{ij} \varepsilon_{ij}+b_{ij} \gamma_{ij}+ c_{ijk}\chi_{ijk}+\beta \theta , \\& T_{0}\dot{\eta }=Q-q_{i,i}, \end{aligned}$$

and the following symmetry relations are assumed to hold in the domain Ω:

15 $$\begin{aligned} C_{ijmn}=C_{mnij},\quad \quad B_{ijmn}=B_{mnij},\qquad A_{ijkmnr}=A_{mnrijk}. \end{aligned}$$

Also, from (12) we deduce the dissipative inequality

16 $$\begin{aligned} \int_{\Omega }\frac{q_{i}\theta_{,i}}{T_{0}}\,dV\le 0, \end{aligned}$$

which leads to

17 $$\begin{aligned} \int_{\Omega }\frac{q_{i,i}\theta }{T_{0}}\,dV- \int_{\partial \Omega }\frac{q \theta }{T_{0}}\,dA \ge 0, \end{aligned}$$

which, obviously, becomes

$$\begin{aligned} \int_{\Omega }\frac{q_{i,i}\theta }{T_{0}}\,dV\ge 0, \end{aligned}$$

when there is no flux, that is, $q=0$.

Let us denote by $t_{\alpha }$ the phase-lag of the thermal displacement gradient, by $t_{q}$ the phase-lag of the heat flux, by $t_{T}$ the phase-lag of the temperature gradient, by $k_{ij}$ the thermal conductivity tensor and by $k_{ij}^{*}$ the conductivity rate tensor. As is enshrined in the literature (see, for instance, [5]), for the heat flux vector in the three-phase-lag theory, we have the following constitutive equation:

$$\begin{aligned} q_{i} ( x, t+t_{q} ) = - \bigl[ k_{ij} \theta_{,j} ( x, t+t _{T} ) +k_{ij}^{*} \alpha_{,j} ( x, t+t_{\alpha } ) \bigr] , \end{aligned}$$

tensors $k_{ij}$ and $k_{ij}^{*}$ being symmetric.

If we develop here in the Taylor series and retain terms of the order $t_{q}^{2}$, we obtain the following equation:

18 $$\begin{aligned}& q_{i}+t_{q}\dot{q}_{i}+ \frac{1}{2}t_{q}^{2}\ddot{q}_{i} \\& \quad =- \bigl[ k_{ij} \theta_{,j}+k_{ij} t_{T}\dot{ \theta }_{,j}+k_{ij}^{*} t_{T} \alpha _{,j} +k_{ij}^{*} t_{\alpha }\dot{\alpha }_{,j} \bigr] ,\quad 0< t_{\alpha }\le t_{T}\le t_{q}. \end{aligned}$$

It is a usual notation $\hat{f}=\int_{0}^{t}f(x,s)\,ds$ with which we introduce two new variables

19 $$\begin{aligned} \xi_{i}=\beta_{i}+t_{T}\dot{ \beta }_{i}, \qquad \varsigma_{i}= \hat{\beta }_{i}+t_{\alpha } \beta_{i} \end{aligned}$$

in order to get a more simple form of equation (18), namely

20 $$\begin{aligned} ( 1+D_{t} ) q_{i}=- \bigl( k_{ij} \dot{\xi }_{j}+k_{ij}^{*} \dot{\varsigma }_{j} \bigr) , \end{aligned}$$

where the differential operator $D_{t}$ is defined by

$$ D_{t}=t_{q}\frac{\partial }{\partial t}+\frac{1}{2}t_{q}^{2} \frac{ \partial^{2}}{\partial t^{2}}. $$

Using the notation $h_{i}=T_{0}^{-1}\hat{q}_{i}$, we can introduce the entropy flux vector *h* by

21 $$\begin{aligned} h=h_{i}n_{i}=T_{0}^{-1}\hat{q}_{i} n_{i}. \end{aligned}$$

Considering the definition of convolution product

$$ (v*w) (t)= \int _{0}^{t}v(x, t-s)w(x,s)\,ds, $$

the energy equation (14)_5_ receives the form

22 $$\begin{aligned} t* ( \eta +h_{i,i,}-R ) =0,\quad R=\frac{1}{T_{0}}{ \hat{Q}}+\eta ^{0}, \end{aligned}$$

where *Q* is the intensity of applied heat source per unit volume and $\eta^{0}$ is the initial value of entropy.

Now we will add the initial and boundary conditions. So, on the domain Ω̄, we consider the initial conditions

23 $$ \begin{aligned} &u_{i}(x,0)=u_{i}^{0}(x),\qquad \dot{u}_{i}(x,0)=u_{i}^{1}(x), \qquad \varphi _{ij}(x,0)=\varphi_{ij}^{0}(x), \\ &\dot{\varphi }_{ij}(x,0)=\varphi_{ij}^{1}(x),\qquad \theta (x,0)=\theta ^{0}(x), \qquad \dot{\theta }(x,0)=\theta^{1}(x), \end{aligned} $$

while on the cylinder $\partial \Omega \times (0,\infty )$ we add the boundary conditions

24 $$ \begin{aligned} &u_{i}(x,t)=\tilde{u}_{i}, \quad (x,t)\in \partial \Omega_{u}\times (0, \infty ), \\ &( \tau_{ij}+ \sigma_{ij} ) (x,t)n_{j}=\tilde{t} _{i}, \quad (x,t)\in \partial \Omega_{u}^{c}\times (0,\infty ), \\ &\varphi_{ij}(x,t)=\tilde{\varphi }_{ij}, \quad (x,t)\in \partial \Omega_{\varphi }\times (0,\infty ), \\ &\mu_{ijk}(x,t)n_{i}= \tilde{m} _{jk}, \quad (x,t)\in \partial \Omega_{\varphi }^{c} \times (0,\infty ), \\ &\alpha (x,t)=\tilde{\alpha }, \quad (x,t)\in \partial \Omega_{\alpha } \times (0, \infty ), \\ & q_{i}(x,t)n_{i}=\tilde{q}, \quad (x,t)\in \partial \Omega_{\alpha }^{c}\times (0,\infty ), \end{aligned} $$

where the surfaces $\partial \Omega_{u}$, $\partial \Omega_{\varphi }$, $\partial \Omega_{\alpha }$ and their complements $\partial \Omega _{u}^{c}$, $\partial \Omega_{\varphi }^{c}$, $\partial \Omega_{\alpha }^{c}$ are subsets of the boundary surface *∂*Ω which satisfy the properties

$$\begin{aligned}& \partial \bar{\Omega }_{u}\cup \partial \Omega_{u}^{c}= \partial \bar{ \Omega }_{\varphi }\cup \partial \Omega_{\varphi }^{c}= \partial \bar{ \Omega }_{\alpha }\cup \partial \Omega_{\alpha }^{c}= \partial \Omega , \\& \partial \Omega_{u}\cap \partial \Omega_{u}^{c}= \partial \Omega_{ \varphi }\cap \partial \Omega_{\varphi }^{c}= \partial \Omega_{\alpha }\cap \partial \Omega_{\alpha }^{c}= \emptyset . \end{aligned}$$

In (23) the functions $u_{i}^{0}(x)$, $u_{i}^{1}(x)$, $\varphi_{ij} ^{0}(x)$, $\varphi_{ij}^{1}(x)$, $\theta^{0}(x)$ and $\theta^{1}(x)$ are prescribed in their domain of definition. Also, in (24) $\tilde{u} _{i}$, $\tilde{t}_{i}$, $\tilde{\varphi }_{ij}$, $\tilde{m}_{jk}$, *α̃* and *q̃* are given functions in their domain of definition and, as above, $n_{i}$ are the components of the outward unit normal to the surface *∂*Ω.

Now we apply the convolution product in (8)_2_ and (8)_3_ so that by using the initial conditions (23) and notations

$$ F_{i}=t*f_{i}+tu_{i}^{1}+u_{i}^{0},\qquad G_{jk}=t*g_{jk}+I_{js} \bigl( t \varphi_{ks}^{1}+\varphi_{ks}^{0} \bigr) $$

we are led to

25 $$\begin{aligned}& t* ( \tau_{ij}+\sigma_{ij} ) _{,j}+ \varrho F_{i}=\varrho u _{i}, \\& t*\mu_{ijk,i}+t*\sigma_{jk}+\varrho G_{jk}=I_{js} \varphi_{ks}. \end{aligned}$$

From (20) and (21) we obtain the equation

$$ h_{i}+t_{q}\dot{h}_{i}+\frac{1}{2}t_{q}^{2} \ddot{h}_{i}= \frac{1}{T _{0}} \bigl( k_{ij} \xi_{j}+k_{ij}^{*}\varsigma_{j} \bigr) , $$

which can be rewritten in the form

26 $$\begin{aligned} ( 1+D_{t} ) h_{i}=- \frac{1}{T_{0}} \bigl( k_{ij}\beta_{j}+k _{ij}t_{T} \dot{\beta }_{j}+k_{ij}^{*}\hat{\beta }_{j}+k_{ij}^{*}t _{\alpha } \beta_{j} \bigr) . \end{aligned}$$

Now, we take into account (14)_4_ and (14)_5_ so that (26) receives the form

27 $$\begin{aligned} ( 1+D_{t} ) \biggl( \beta \dot{\theta }+a_{ij} \dot{\varepsilon }_{ij}+b_{ij}\dot{\gamma }_{ij}+c_{ijk}\dot{\chi } _{ijk}-\frac{Q}{T_{0}} \biggr) = \frac{1}{T_{0}} \bigl( k_{ij} \dot{\xi }_{j}+k_{ij}^{*} \dot{\varsigma }_{j} \bigr) , \end{aligned}$$

which is known as the heat transport equation.

If we integrate on the domain Ω in (27), we are led to

28 $$\begin{aligned}& \int_{\Omega } \theta \biggl[ D_{t} ( \beta \dot{\theta } + a_{ij}\dot{\varepsilon }_{ij} + b_{ij}\dot{\gamma }_{ij} + c _{ijk}\dot{\chi }_{ijk} ) - ( 1 + D_{t} ) \frac{Q}{T _{0}} - \frac{1}{T_{0}} \bigl( k_{ij} \dot{\xi }_{j} + k_{ij}^{*} \dot{\varsigma }_{j} \bigr) _{,i} \biggr]\,dV \\& \quad {}=-\int_{\Omega }\theta ( \beta \dot{\theta }+a_{ij} \dot{ \varepsilon }_{ij}+b_{ij}\dot{\gamma }_{ij}+c_{ijk} \dot{\chi } _{ijk} )\,dV, \end{aligned}$$

which, using relation (14)_4_, receives the simpler form

29 $$\begin{aligned} \int_{\Omega }\theta \biggl( \dot{\eta }-\frac{Q}{T_{0}} \biggr)\,dV= \int_{\Omega }\theta \biggl[ \frac{1}{T_{0}} \bigl( k_{ij}\dot{\xi } _{j} + k_{ij}^{*}\dot{ \varsigma }_{j} \bigr) _{,i}+ D_{t} \biggl( \frac{Q}{T _{0}}-\dot{\eta } \biggr) \biggr]\,dV. \end{aligned}$$

Summarizing, the mixed initial boundary value problem for the three-phase-lag dipolar thermoelastic bodies, denoted by ${\mathcal {P}}$, is given by the geometric equations (1), the constitutive equations (14), (18) and (22), the equation of motion (25), the initial conditions (23) and the boundary conditions (24).

## Main results

First, we require the elasticity tensors occurring in (13) and (14) to satisfy the following condition: there is a constant $c_{0}>0$ so that

30 $$\begin{aligned}& C_{ijmn}u_{ij}u_{mn}+ 2G_{ijmn}u_{ij}v_{mn}+ 2F_{ijmnr}u_{ij}w_{mnr}+ B_{ijmn}v_{ij}v_{mn} \\& \quad {}+2D_{ijmnr}v_{ij} w_{mnr}+ A_{ijkmnr}w_{ijk}w_{mnr} \ge c_{0} ( u _{ij}u_{ij}+v_{ij}v_{ij}+w_{ijk}w_{ijk} ) \end{aligned}$$

for any three tensors $u_{ij}, v_{ij}$ and $w_{ijk}$.

A result regarding the uniqueness of solution to the problem ${\mathcal {P}}$ is given in the next theorem.

### Theorem 1

*Assume that*

(i) $\varrho (x)>0, \beta (x)>0, T_{0}(x)>0, \forall x\in \Omega $;
(ii) $I_{ij}(x)$ *is a positive definite tensor*;
(iii) *condition* (30) *takes place*.

*Then the problem* ${\mathcal {P}}$ *has at most one solution*.

### Proof

Suppose, by contradiction, that the mixed problem ${\mathcal {P}}$ would have two solutions $( u_{i}^{(1)},\varphi_{ij} ^{(1)},\theta^{(1)} ) $ and $( u_{i}^{(2)},\varphi_{ij}^{(2)}, \theta^{(2)} ) $. Because of linearity, their difference is also a solution to the problem ${\mathcal {P}}$. Let us use the notation $u_{i}^{(d)}=u_{i}^{(2)}-u_{i}^{(1)}$, $\varphi_{ij}^{(d)}=\varphi _{ij}^{(2)}-\varphi_{ij}^{(1)}$, $\theta^{(d)}=\theta^{(2)}-\theta ^{(1)}$. Of course, $( u_{i}^{(d)},\varphi_{ij}^{(d)},\theta^{(d)} ) $ satisfies the problem ${\mathcal {P}}$ in the particular case when all the elements of external data are zeros, the initial data are null and also with null boundary conditions. To obtain an estimate of the difference $( u_{i}^{(d)},\varphi_{ij}^{(d)},\theta^{(d)} ) $, we will use the integral

$$ \int_{\Omega } ( \tau_{ij}\dot{\varepsilon }_{ij}+\sigma_{ij} \dot{\gamma }_{ij}+ \mu_{ijk}\dot{\chi }_{ijk} )\,dV. $$

Here and until the end of the demonstration, because there is no likelihood of confusion, we give up writing the superscript $^{(d)}$ for all functions.

Using the equations of motion (8)_2_ and (8)_3_ for the difference solutions and applying the divergence theorem, because of null boundary conditions, we are led to

31 $$\begin{aligned} \int_{\Omega } ( \tau_{ij}\dot{\varepsilon }_{ij}+\sigma_{ij} \dot{\gamma }_{ij}+ \mu_{ijk}\dot{\chi }_{ijk} )\,dV=- \int_{ \Omega } ( \varrho \ddot{u}_{i} \dot{u}_{i}+I_{jk}\ddot{\varphi } _{js}\dot{\varphi }_{ks} )\,dV. \end{aligned}$$

This identity can be rewritten in the form

32 $$\begin{aligned}& \frac{1}{2} \frac{d}{dt} \biggl[ \int_{\Omega } \bigl( C_{ijmn} \varepsilon_{ij} \varepsilon_{mn}+ 2G_{ijmn}u\varepsilon_{ij}\gamma _{mn}+ 2F_{ijmnr}\varepsilon_{ij}\chi_{mnr} \\& \quad {}+B_{ijmn}\gamma_{ij}\gamma_{mn}+ 2D_{ijmnr} \gamma_{ij} \chi_{mnr}+ A _{ijkmnr}\chi_{ijk} \chi_{mnr} \\& \quad {} +\varrho \dot{u}_{i}\dot{u}_{i}+I_{jk} \dot{\varphi }_{js} \dot{\varphi }_{ks}+\beta \theta^{2} \bigr)\,dV \biggr] = \int_{\Omega} \dot{\eta }\theta \,dV \end{aligned}$$

upon adding $\beta \theta \dot{\theta }$ and considering the constitutive equations (14) and the symmetry relations (15).

On the other hand, taking into account inequality (17) in the particular case $q=0$ and the constitutive relation (14)_5_, we obtain

33 $$\begin{aligned} \int_{\Omega }\frac{1}{T_{0}}q_{i,i}\theta \,dV=- \int_{\Omega } \dot{\eta }\theta \ge 0, \end{aligned}$$

and this inequality with (32) lead to

34 $$\begin{aligned}& \frac{1}{2} \frac{d}{dt} \biggl\{ \int_{\Omega } \bigl( C_{ijmn} \varepsilon_{ij} \varepsilon_{mn}+ 2G_{ijmn}u\varepsilon_{ij}\gamma _{mn}+ 2F_{ijmnr}\varepsilon_{ij}\chi_{mnr} \\& \quad {}+B_{ijmn}\gamma_{ij}\gamma_{mn}+ 2D_{ijmnr} \gamma_{ij} \chi_{mnr}+ A _{ijkmnr}\chi_{ijk} \chi_{mnr} \\& \quad {}+\varrho \dot{u}_{i}\dot{u}_{i}+I_{jk} \dot{\varphi }_{js} \dot{\varphi }_{ks}+\beta \theta^{2} \bigr)\,dV\frac{}{} \biggr\} \le 0 \end{aligned}$$

for all $(x,t)\in \Omega \times [0, \infty )$.

Clearly, from (34) it easy to obtain $\dot{u}_{i}=0$, $\dot{\varphi } _{ij}=0$ and $\theta =0$. As such, if we consider the fact that for difference of solutions, the initial conditions are null, we deduce $u_{i}=0$ and $\varphi_{ij}=0$, which concludes the proof of the theorem. □

Let us consider two different systems of external data acting on our dipolar body, namely

35 $$\begin{aligned} {\mathcal {S}}^{(\nu )}&= \bigl\{ F_{i}^{(\nu )} , G_{jk}^{(\nu )} , R^{(\nu )} , \tilde{u}_{i}^{(\nu )} , \tilde{\varphi }_{ij}^{(\nu )} , \tilde{\alpha }^{(\nu )}, \\ &\quad {}\tilde{t}_{i}^{(\nu )} , \tilde{m}_{ij} ^{(\nu )} , \tilde{q}^{(\nu )} , u_{i}^{0(\nu )} , u_{i}^{1(\nu )} , \varphi_{ij}^{0(\nu )} , \varphi_{ij}^{1(\nu )} , \theta^{0( \nu )} , \theta^{1(\nu )} \bigr\} , \end{aligned}$$

where $\nu =1,2$. The solutions of mixed problem corresponding to each system of external data will be denoted by $s^{(\nu )}$, that is,

$$ s^{(\nu )}= \bigl\{ u_{i}^{(\nu )}, \varphi_{ij}^{(\nu )}, \theta^{( \nu )}, \alpha^{(\nu )} \bigr\} , \quad \nu =1,2. $$

The link between charging systems ${\mathcal {S}}^{(\nu )}$ and corresponding solutions $s^{(\nu )}$ is given in the following theorem.

### Theorem 2

*Between loading systems* ${\mathcal {S}}^{(\nu )}$ *and corresponding solutions* $s^{(\nu )}$, *the next reciprocal relation of Betti type holds*

36 $$\begin{aligned}& \int_{\Omega } \biggl( \varrho F_{i}^{(1)}*u_{i}^{(2)}+ \varrho G_{ij} ^{(1)}*\varphi_{ij}^{(2)}-t*R^{(1)}* \theta^{(2)}-\frac{1}{T_{0}}t*q _{i}^{(1)}* \beta_{i}^{(2)} \biggr)\,dV \\& \qquad {}+ \int_{\partial \Omega } \biggl( t*t_{i}^{(1)}*u_{i}^{(2)}+t*m_{ij}^{(1)}* \varphi_{ij}^{(2)}+\frac{1}{T_{0}}t*q^{(1)}* \alpha^{(2)} \biggr)\,dA \\& \quad = \int_{\Omega } \biggl( \varrho F_{i}^{(2)}*u_{i}^{(1)}+ \varrho G_{ij} ^{(2)}*\varphi_{ij}^{(1)}-t*R^{(2)}* \theta^{(1)}-\frac{1}{T_{0}}t*q _{i}^{(2)}* \beta_{i}^{(1)} \biggr)\,dV \\& \qquad {}+ \int_{\partial \Omega } \biggl( t_{i}^{(2)}*u_{i}^{(1)}+m_{ij}^{(2)}* \varphi_{ij}^{(1)}+\frac{1}{T_{0}}t*q^{(2)}* \alpha^{(1)} \biggr)\,dA. \end{aligned}$$

### Proof

With the help of constitutive equation (14)_1_ and symmetry relations (15), we can write

37 $$\begin{aligned}& \int_{\Omega } \bigl( t*\tau_{ij}^{(1)}* \varepsilon_{ij}^{(2)}-t*\tau _{ij}^{(2)}* \varepsilon_{ij}^{(1)} \bigr)\,dV \\& \quad = \int_{\Omega } \bigl( t*G_{ijmn}\gamma_{mn}^{(1)}* \varepsilon_{ij} ^{(2)}+t*F_{ijmnr}\chi_{mnr}^{(1)}* \varepsilon_{ij}^{(2)}- t*a_{ij} \theta^{(1)}\varepsilon_{ij}^{(2)} \bigr)\,dV \\& \quad \quad {}- \int_{\Omega } \bigl( t*G_{ijmn}\gamma_{mn}^{(2)}* \varepsilon_{ij}^{(1)}+t*F _{ijmnr}\chi_{mnr}^{(2)}* \varepsilon_{ij}^{(1)}- t*a_{ij}\theta^{(2)} \varepsilon_{ij}^{(1)} \bigr)\,dV. \end{aligned}$$

Also, with (14)_2_ and symmetry relations (15), we can write

38 $$\begin{aligned}& \int_{\Omega } \bigl( t*\sigma_{ij}^{(1)}* \gamma_{ij}^{(2)}-t*\sigma _{ij}^{(2)}* \gamma_{ij}^{(1)} \bigr)\,dV \\& \quad = \int_{\Omega } \bigl( t*G_{ijmn}\gamma_{ij}^{(1)}* \varepsilon_{mn} ^{(2)}+t*D_{ijmnr}\chi_{mnr}^{(1)}* \gamma_{ij}^{(2)}- t*b_{ij}\theta ^{(1)} \gamma_{ij}^{(2)} \bigr)\,dV \\& \quad\quad {}-\int_{\Omega } \bigl( t*G_{ijmn}\gamma_{mn}^{(2)}* \varepsilon_{ij}^{(1)}+t*D _{ijmnr}\chi_{mnr}^{(2)}* \gamma_{ij}^{(1)}- t*a_{ij}\theta^{(2)} \varepsilon_{ij}^{(1)} \bigr)\,dV. \end{aligned}$$

Similarly, with the help of (14)_3_ and symmetry relations (15), we can write

39 $$\begin{aligned}& \int_{\Omega } \bigl( t*\mu_{ijk}^{(1)}* \chi_{ijk}^{(2)}-t*\mu_{ijk} ^{(2)}* \chi_{ijk}^{(1)} \bigr)\,dV \\& \quad = \int_{\Omega } \bigl( t*F_{ijkmn}\chi_{ijk}^{(1)}* \varepsilon_{mn} ^{(2)}+t*D_{ijkmn}\chi_{ijk}^{(1)}* \gamma_{mn}^{(2)}- t*c_{ijk} \theta^{(1)} \chi_{ijk}^{(2)} \bigr)\,dV \\& \quad \quad {}- \int_{\Omega } \bigl( t*F_{ijkmn}\chi_{ijk}^{(2)}* \varepsilon_{mn} ^{(1)}+t*D_{ijkmn}\chi_{ijk}^{(2)}* \gamma_{mn}^{(1)}- t*c_{ijk} \theta^{(2)} \chi_{ijk}^{(1)} \bigr)\,dV. \end{aligned}$$

Now, we will use the constitutive equation for entropy, (14)_4_, in order to obtain

40 $$\begin{aligned}& \int_{\Omega } \bigl( t*\eta^{(1)}*\theta^{(2)}-t* \eta^{(2)}*\theta ^{(1)} \bigr)\,dV \\& \quad = \int_{\Omega } \bigl( t*a_{ij} \varepsilon_{ij}^{(1)}* \theta^{(2)}+t*b _{ij}\dot{\gamma }_{ij}^{(1)}* \theta^{(2)}+ t*c_{ijk}\dot{\chi }_{ijk} ^{(1)}*\theta^{(2)} \bigr)\,dV \\& \quad \quad {}- \int_{\Omega } \bigl( t*a_{ij} \varepsilon_{ij}^{(2)}* \theta^{(1)}+t*b _{ij}\dot{\gamma }_{ij}^{(2)}* \theta^{(1)}+ t*c_{ijk}\dot{\chi }_{ijk} ^{(2)}*\theta^{(1)} \bigr)\,dV. \end{aligned}$$

Finally, if we add member by member equalities (37), (38) and (39) and then subtract equality (40), we obtain

$$\begin{aligned}& \int_{\Omega } \bigl( t*\tau_{ij}^{(1)}* \varepsilon_{ij}^{(2)}-t*\tau _{ij}^{(2)}* \varepsilon_{ij}^{(1)} +t*\sigma_{ij}^{(1)}* \gamma_{ij} ^{(2)}-t*\sigma_{ij}^{(2)}* \gamma_{ij}^{(1)} \\& \quad {}+t*\mu_{ijk}^{(1)}*\chi_{ijk}^{(2)}-t* \mu_{ijk}^{(2)}*\chi_{ijk} ^{(1)} -t* \eta^{(1)}*\theta^{(2)}+t*\eta^{(2)}* \theta^{(1)} \bigr)\,dV=0, \end{aligned}$$

and, obviously, this equality can be rewritten in the form

41 $$\begin{aligned}& \int_{\Omega } \bigl( t*\tau_{ij}^{(1)}* \varepsilon_{ij}^{(2)} +t* \sigma_{ij}^{(1)}* \gamma_{ij}^{(2)} +t*\mu_{ijk}^{(1)}* \chi_{ijk}^{(2)} -t*\eta^{(1)}*\theta^{(2)} \bigr)\,dV \\& \quad = \int_{\Omega } \bigl( t*\tau_{ij}^{(2)}* \varepsilon_{ij}^{(1)} +t* \sigma_{ij}^{(2)}* \gamma_{ij}^{(1)} +t*\mu_{ijk}^{(2)}* \chi_{ijk}^{(1)} -t*\eta^{(2)}*\theta^{(1)} \bigr)\,dV. \end{aligned}$$

In (41) we will consider the equations of motion (8)_2_, (8)_3_, the energy equation (22) and the kinetic relations (1). Then we apply the divergence theorem and the boundary conditions (24) so that we are led to the identity

42 $$\begin{aligned}& \int_{\Omega } \bigl( \varrho F_{i}^{(1)}*u_{i}^{(2)}+ \varrho G_{ij} ^{(1)}*\varphi_{ij}^{(2)}-t* R^{(1)}*\theta^{(2)}-t* h_{i}^{(1)}* \theta_{,i}^{(2)} \bigr)\,dV \\& \qquad {}+ \int_{\partial \Omega } \bigl( t*t_{i}^{(1)}*u_{i}^{(2)}+t*m_{ij}^{(1)}* \varphi_{ij}^{(2)}+t*h^{(1)}*\theta^{(2)} \bigr)\,dA \\& \quad = \int_{\Omega } \bigl( \varrho F_{i}^{(2)}*u_{i}^{(1)}+ \varrho G_{ij} ^{(2)}*\varphi_{ij}^{(1)}-t* R^{(2)}*\theta^{(1)}-t* h_{i}^{(2)}* \theta_{,i}^{(1)} \bigr)\,dV \\& \qquad {}+ \int_{\partial \Omega } \bigl( t*t_{i}^{(2)}*u_{i}^{(1)}+t*m_{ij}^{(2)}* \varphi_{ij}^{(1)}+t*h^{(2)}*\theta^{(1)} \bigr)\,dA. \end{aligned}$$

Considering that $h_{i}=\frac{1}{T_{0}}\hat{q}_{i}$, $\alpha = \hat{\theta }$ and $\dot{\beta }_{i}=\theta_{,i}$, from (42) we immediately obtain the desired identity (36). □

### Remark

We want to give an explicit form for the components of the heat flux vector $q_{i}$. So, if we integrate in (20) and take into account the initial conditions $q_{i}(0)=0$ and $\frac{\partial }{ \partial t}q_{i}(0)=0$, we will obtain

43 $$\begin{aligned} q_{i}=- \bigl( k_{ij}a_{j}+k_{ij}^{*}b_{j} \bigr) , \end{aligned}$$

in which, we remind that $k_{ij}$ is the thermal conductivity tensor and $k_{ij}^{*}$ is the conductivity rate tensor. For $a_{j}$ and $b_{j}$, we obtain the expressions

$$\begin{aligned}& a_{j}=\frac{2e^{-t/\tau_{q}}}{\tau_{q}} \biggl[ \sin \frac{t}{\tau_{q}} \int_{0}^{t} \biggl( e^{\frac{t}{\tau_{q}}}\cos \frac{t}{\tau_{q}} \dot{\xi }_{j} \biggr)\,d\tau_{q} - \cos \frac{t}{\tau_{q}} \int_{0}^{t} \biggl( e^{\frac{t}{\tau_{q}}}\sin \frac{t}{\tau_{q}}\dot{\xi }_{j} \biggr)\,d\tau_{q} \biggr], \\& b_{j}=\frac{2e^{-t/\tau_{q}}}{\tau_{q}} \biggl[ \cos \frac{t}{\tau_{q}} \int_{0}^{t} \biggl( e^{\frac{t}{\tau_{q}}}\sin \frac{t}{\tau_{q}} \dot{\varsigma }_{j} \biggr)\,d\tau_{q} - \sin \frac{t}{\tau_{q}} \int _{0}^{t} \biggl( e^{\frac{t}{\tau_{q}}}\cos \frac{t}{\tau_{q}} \dot{\varsigma }_{j} \biggr)\,d\tau_{q} \biggr] , \end{aligned}$$

where *ξ* and $\varsigma_{j}$ are defined in (19) and $\tau_{q}$ is the phase-lag of the heat flux.

Other important result of our paper is a variational principle. We strengthen the known variational principle in order to cover the three-phase-lag dipolar thermoelasticity theory.

Assuming that the tensors $k_{ij}$ and $k_{ij}^{*}$ can be reversed, we will use the symmetric tensors $\lambda_{ij}$ and $\lambda_{ij}^{*}$, defined by

44 $$\begin{aligned} \lambda_{ij}= [ k_{ij} ] ^{-1},\qquad \lambda_{ij}^{*}= \bigl[ k _{ij}^{*} \bigr] ^{-1}. \end{aligned}$$

We will write $h_{i}$ in the form

45 $$\begin{aligned} h_{i}=h_{i}^{(I)}+h_{i}^{(II)}+h_{i}^{(III)}+h_{i}^{(IV)} \end{aligned}$$

so that considering (18) and (26) we obtain

46 $$ \begin{aligned} &( 1+D_{t} ) \lambda_{ij}h_{j}^{(I)}+ \frac{1}{T_{0}}\beta _{i}=0, \qquad ( 1+D_{t} ) \lambda_{ij}h_{j}^{(II)}+\frac{\tau _{T}}{T_{0}} \theta_{,i}=0, \\ &( 1+D_{t} ) \lambda_{ij}^{*}s_{j}^{(III)}+ \frac{1}{T_{0}} \beta_{i}=0, \qquad ( 1+D_{t} ) \lambda_{ij}^{*}h_{j}^{(IV)}+ \frac{ \tau_{\alpha }}{T_{0}}\beta_{i}=0, \end{aligned} $$

where $\beta_{i}=\hat{\theta }_{,i}$ and $s_{i}^{(III)}=\partial h _{i}^{(III)}/\partial t$.

Motivation of the decomposition (45) will appear later. We will call an admissible process be an ordered array

47 $$\begin{aligned} p= ( u_{i}, \varphi_{ij}, \alpha , \theta , \varepsilon_{ij}, \gamma_{ij}, \chi_{ijk}, \tau_{ij}, \sigma_{ij}, \beta_{i}, \eta , h _{i}, h_{i,i}, q_{i} ) \end{aligned}$$

having as components sufficiently regular functions on their domain of definition.

Let us denote by ${\mathcal {A}}$ the set of all admissible processes which is a linear space with addition and scalar multiplication.

On ${\mathcal {A}}$ and for each $t\in [0,\infty )$, we define the functional ${\mathcal {F}}_{t}(p)$ by

48 $$\begin{aligned} {\mathcal {F}}_{t}(p)= {}&\frac{1}{2} \int_{\Omega }t* ( C_{ijmn}\varepsilon _{mn}* \varepsilon_{ij}+2G_{ijmn}\varepsilon_{mn}* \gamma_{ij} +2F_{ijmnr} \chi_{mnr}*\varepsilon_{ij} \\ &{} +B_{ijmn}\gamma_{mn}*\gamma_{ij} +2D_{ijmnr}\chi_{mnr}*\gamma _{ij}+A_{ijkmnr} \chi_{mnr}*\chi_{ijk} )\,dV \\ &{}+ \int_{\Omega } \bigl[ \varrho u_{i}*u_{i}+I_{jk} \varphi_{js}*\varphi _{ks}-t*(\eta -R)*\theta \bigr]\,dV \\ &{}+\int_{\Omega } \frac{1}{2\beta } \bigl[ t * ( \eta - a_{ij}\varepsilon_{ij} - b_{ij} \gamma_{ij} - c_{ijk}\chi_{ijk} ) \\ &{}* ( \eta - a_{mn}\varepsilon_{mn} - b_{mn} \gamma_{mn} - c_{mnr}\chi_{mnr} ) \bigr]\,dV \\ &{}-\int_{\Omega } \bigl\{ \bigl[ t* ( \tau_{ij}+ \sigma_{ij} ) _{,j}+\varrho F_{i} \bigr] *u_{i}+t*\tau_{ij}*\varepsilon_{ij} +t* \sigma_{ij}*\gamma_{ij} \bigr\} \,dV \\ & {}- {\int _{\Omega } \bigl[ ( t*\mu _{ijk,i}+t*\sigma _{jk}+\varrho G_{jk} ) *\varphi _{jk}-t*\mu _{ijk}*\chi _{ijk} \bigr]\,dV} \\ & {}+\frac{1}{2}\int_{\Omega } ( 1 + D_{t} ) \biggl( \hat{T}_{0} \lambda_{ij}h_{i}^{(I)} * h_{j}^{(I)} + t * \frac{T_{0}}{\tau_{T}}\lambda_{ij}h_{i}^{(II)} * h_{j} ^{(II)} \\ & {}+ \lambda_{ij}^{*}q_{i}^{(III)} * h_{j}^{(III)} + \hat{\frac{T_{0}}{\tau _{\alpha }}}\lambda_{ij}^{*}h_{i}^{(IV)} * h_{j}^{(IV)} \biggr)\,dV \\ & {}+\int_{\Omega } \biggl( \hat{h}_{i}*\beta_{i}+ \hat{h}_{i}*\alpha_{,i}-t*\frac{1}{T_{0}}q_{i}* \beta_{i}+\hat{h}_{i,i}*\alpha - \frac{1}{T_{0}}t*\hat{q} _{i,i}*\theta \biggr)\,dV \\ & {}+\int_{\partial \Omega_{u}} ( t*t_{i}*\tilde{u}_{i} )\,dA + \int_{\partial \Omega_{u}^{c}} \bigl[ t* ( t_{i}-\tilde{t}_{i} ) *u _{i} \bigr]\,dA+\int_{\partial \Omega_{\varphi }} ( t* m_{ij}* \tilde{\varphi }_{ij} )\,dA \\ & {}+\int_{\partial \Omega_{\varphi }^{c}} \bigl[ t* ( m_{ij}- \tilde{m}_{ij} ) *\varphi_{ij} \bigr]\,dA- \int_{\partial \Omega_{\alpha }} \bigl[ \hat{h}* ( \alpha - \tilde{\alpha } ) \bigr]\,dA-\int_{\partial \Omega_{\alpha } ^{c}} [ \hat{\tilde{h}}*\alpha ]\,dA. \end{aligned}$$

Now, we can state and prove the convolutional variational principle for the three-phase-lag dipolar thermoelasticity theory.

### Theorem 3

*If the symmetric tensors* $k_{ij}$ *and* $k_{ij}^{*}$ *can be reversed*, $\tau_{\alpha }>0$, $\tau_{T}>0$ *and the symmetry relations* (15) *hold on* Ω, *then*

49 $$\begin{aligned} \delta {\mathcal {F}}_{t}(p)=0,\quad t\ge 0 \end{aligned}$$

*if and only if* *p* *is a solution of the mixed initial boundary value problem* $\mathcal {P}$.

### Proof

First, we will prove the inverse implication, that is, assuming that *p* from (47) is a solution of the mixed problem ${\mathcal {P}}$, we must prove that $\delta {\mathcal {F}}_{t}(p)=0$. Along with the admissible process *p*, we consider another process

$$ \breve{p}= ( \breve{u}_{i}, \breve{\varphi }_{ij}, \breve{ \alpha }, \breve{\theta }, \breve{\varepsilon }_{ij}, \breve{\gamma }_{ij}, \breve{\chi }_{ijk}, \breve{\tau }_{ij}, \breve{\sigma }_{ij}, \breve{\beta }_{i}, \breve{\eta }, \breve{h} _{i}, \breve{h}_{i,i}, \breve{q}_{i} ) . $$

Of course, we have

$$ p, \breve{p} \in {\mathcal {A}}\quad \Rightarrow\quad p+\kappa \breve{p} \in {\mathcal {A}}, \quad \forall \kappa \in R. $$

Let us compute the variation of the functional ${\mathcal {F}}_{t}(p)$

50 $$\begin{aligned} \delta {\mathcal {F}}_{t}(p)={}& \int_{\Omega } \biggl\{ t* \biggl[ C_{ijmn}\varepsilon_{mn}+G_{ijmn}\gamma_{mn}+F_{ijmnr}\chi_{mnr} \\ & {}- \frac{a_{ij}}{\beta } ( \eta -a_{mn}\varepsilon_{mn}-b_{mn}\gamma_{mn}-c_{ijk}\chi_{ijk} ) -\tau_{ji} \biggr] * \breve{\varepsilon }_{ij} \\ &{}+t* \biggl[ G_{ijmn}\varepsilon_{mn}+B_{ijmn}\gamma_{mn}+D_{ijmnr} \chi_{mnr} \\ & {}-\frac{b_{ij}}{\beta } ( \eta -a_{mn}\varepsilon_{mn}-b_{mn}\gamma_{mn}-c_{ijk}\chi_{ijk} ) -\sigma_{ji} \biggr] *\breve{\gamma }_{ij} \\ & {}+ t* \biggl[ F_{ijkmn}\varepsilon_{mn}+D_{mnijk} \gamma_{mn}+A_{ijkmnr} \chi_{mnr} \\ & {} - \frac{c_{ijk}}{\beta } ( \eta -a_{mn}\varepsilon _{mn}-b_{mn}\gamma_{mn}-c_{ijk} \chi_{ijk} ) -\mu_{ijk} \biggr] * \breve{\chi }_{ijk} \biggr\} \,dV \\ & {}+ \int_{\Omega } \biggl\{ t* \biggl[ -\theta +\frac{1}{\beta } ( \eta -a _{mn}\varepsilon_{mn}-b_{mn} \gamma_{mn}-c_{mnr}\chi_{mnr} ) \biggr] * \breve{\eta } \biggr\} \,dV \\ & {}+ \int_{\Omega } \bigl[ t * ( R - \eta - h_{i,i} ) * \breve{ \theta } \bigr]\,dV + \int_{\Omega } \bigl[ \bigl( \varrho u _{i} - t* ( \tau_{ij} + \sigma_{ij} ) _{,j} - \varrho F_{i} \bigr) *\breve{u}_{i} \bigr]\,dV \\ & {}+ \int_{\Omega } \bigl[ ( I_{js}\varphi_{ks}-t* \mu_{ijk,i}-t* \sigma_{jk}-\varrho G_{jk} ) *\breve{ \varphi }_{jk} \bigr]\,dV \\ & {}+ \int_{\Omega } \bigl[ T_{0} ( 1 + D_{t} ) \lambda_{ij}h _{j}^{(I)} + \beta_{i} \bigr] * \breve{h}_{i}^{(I)}\,dV \\ &{} + \int_{\Omega } t * \biggl[ \frac{T_{0}}{\tau_{T}} ( 1 + D_{t} ) \lambda _{ij}h_{j}^{(II)} + \beta_{i} \biggr] * \breve{h}_{i}^{(II)}\,dV \\ & {}+ \int_{\Omega } \bigl[ ( 1 + D_{t} ) \lambda_{ij}^{*}h _{j}^{(III)} + \beta_{i} \bigr]* \breve{h}_{i}^{(III)}\,dV \\ &{}+ \int_{\Omega } t* \biggl[ \frac{T_{0}}{\tau_{\alpha }} ( 1 + D_{t} ) \lambda_{ij}^{*}h_{j}^{(IV)} + \beta_{i} \biggr] * \breve{h}_{i}^{(IV)}\,dV \\ & {}+ \int_{\Omega } \biggl[ \biggl( h_{i} - \frac{q_{i}}{T_{0}} \biggr) * \breve{\alpha }_{,i} + \biggl( h_{i,i} - \frac{q_{i,i}}{T_{0}} \biggr) * \breve{\alpha } \biggr]\,dV \\ &{} + \int_{\Omega } \bigl[ ( \alpha_{,i} - \beta_{i} ) * \breve{h}_{i} + ( \alpha - \hat{\theta } ) * \breve{h}_{i,i} \bigr]\,dV \\ & {}+ \int_{\partial \Omega_{u}} \bigl[ t* ( \tilde{u}_{i}-u_{i} ) * \breve{t}_{i} \bigr]\,dA+ \int_{\partial \Omega_{u}^{c}} \bigl[ t* ( t _{i}-\tilde{t}_{i} ) *\breve{u}_{i} \bigr]\,dA \\ & {}+ \int_{\partial \Omega_{\varphi }} \bigl[ t* ( \tilde{\varphi } _{ij}- \varphi_{ij} ) *\breve{m}_{ij} \bigr]\,dA+\int_{\partial \Omega_{\varphi }^{c}} \bigl[ t* ( m_{ij}-\tilde{m} _{ij} ) *\breve{\varphi }_{ij} \bigr]\,dA \\ & {}+ \int_{\partial \Omega_{\alpha }} \bigl[ t* ( \tilde{\alpha }- \alpha ) *\breve{h} \bigr]\,dA+ \int_{\partial \Omega_{\alpha } ^{c}} \bigl[ t* ( h-\tilde{h} ) *\breve{\alpha } \bigr]\,dA. \end{aligned}$$

Here, to compute the first variation of $\hat{h}_{i}*\beta_{i}$, we have used the above decomposition of $h_{i}$ in four components.

If *p* is a solution of the mixed problem $\mathcal {P}$, then the equations of motion (25), the energy equations (20), the initial conditions (23) and the boundary conditions (24) are satisfied. Also, equations (46) are satisfied. If we take into account these equations and conditions in (50), we obtain $\delta {\mathcal {F}}_{t}(p)=0$.

Now, let us prove the reverse implication, namely, assuming that

51 $$\begin{aligned} \delta {\mathcal {F}}_{t}(p)=0, \quad t\ge 0, \end{aligned}$$

we have to prove that *p* is a solution of the mixed problem $\mathcal {P}$.

To this aim we use a suggestion given by Gurtin in the paper [28]. We take a displacement $\breve{u}_{i}$ such that it and its space derivatives vanish on cylinder $\partial \Omega \times [0,\infty )$ and choose the particular admissible process *p̆* of the form

$$\begin{aligned} \breve{p}= ( \breve{u}_{i}, 0, 0, 0, 0, 0, 0, 0, 0, 0, 0, 0, 0, 0 ). \end{aligned}$$

We substitute *p̆* in (50) such that (51) reduces to

$$ \int_{\Omega } \bigl[ \bigl( t* ( \tau_{ij} + \sigma_{ij} ) _{,j} + \varrho F_{i}-\varrho u_{i} \bigr) *\breve{u}_{i} \bigr]\,dV=0 $$

for arbitrary $\breve{u}_{i}$. According to the fundamental lemma of calculus of variations, we obtain the equation of motion (25)_1_.

Now we take *p̆* of the form (49) but suppose that $\breve{u} _{i}$ vanishes on $\partial \Omega_{u}\times [0,\infty )$. With this *p̆*, (50) and (51) lead to

$$ t* ( t_{i}-\tilde{t}_{i} ) =0 \quad \mbox{on } \partial \Omega_{u}\times [0,\infty ), $$

by using again the fundamental lemma of calculus of variations. This last equality implies the boundary condition (24)_2_.

We repeat the above procedure by making suitable choices of process *p̆*. With each choice of *p̆*, by applying the fundamental lemma of calculus of variations, we get an equation or condition of a mixed problem.

Now we can give a justification for decomposition (45).

So, if we choose $\breve{p}= ( 0, 0, 0, 0, 0, 0, 0, 0, 0, 0, 0, \breve{h}_{i}^{(I)}, 0, 0 ) $, from (50) and (51) we deduce the equation $T_{0} ( 1+D_{t} ) \lambda_{ij}h_{j}^{(I)}+\beta _{i}=0$, which can be rewritten in the form

52 $$\begin{aligned} ( 1+D_{t} ) h_{j}^{(I)}+ \frac{1}{T_{0}}k_{ij}\beta_{i}=0. \end{aligned}$$

Let us consider a process *p̆* of the form $\breve{p}= ( 0, 0, 0, 0, 0, 0, 0, 0, 0, 0, 0, \breve{h}_{i}^{(II)}, 0, 0 ) $. From (49) and (50), with this *p̆*, we obtain the equation $( 1+D_{t} ) \lambda_{ij}h_{j}^{(II)}+\tau_{T}/T_{0}\theta _{,i}=0$ which can be rewritten in the form

53 $$\begin{aligned} ( 1+D_{t} ) h_{j}^{(II)}+ \frac{\tau_{T}}{T_{0}}k_{ij}\theta _{,i}=0. \end{aligned}$$

Next, we choose a process *p̆* of the form $\breve{p}= ( 0, 0, 0, 0, 0, 0, 0, 0, 0, 0, 0, \breve{h}_{i}^{(III)}, 0, 0 ) $ and if we use (49) and (50), with this *p̆*, we are led to the equation $( 1+D_{t} ) \lambda_{ij}^{*}h_{j}^{(III)}+\beta _{i}=0$, or, in another form,

54 $$\begin{aligned} ( 1+D_{t} ) h_{j}^{(III)}+ \frac{1}{T_{0}}k_{ij}^{*} \hat{\beta }_{,i}=0. \end{aligned}$$

Finally, we take the process *p̆* in the form $\breve{p}= ( 0, 0, 0, 0, 0, 0, 0, 0, 0, 0, 0, \breve{h}_{i}^{(IV)}, 0, 0 ) $. Using (49) and (50), with this *p̆*, we obtain the equation $( 1+D_{t} ) \lambda_{ij}^{*}h_{j}^{(IV)}+ \tau_{\alpha }/T_{0}\beta_{i}=0$ which can be rewritten in the form

55 $$\begin{aligned} ( 1+D_{t} ) h_{j}^{(IV)}+ \frac{\tau_{\alpha }}{T_{0}}k_{ij} ^{*}\beta_{i}=0. \end{aligned}$$

By adding relations (52)-(55), we obtain (26), and, in this way, decomposition (45) is justified. The proof of Theorem 1 is completed. □

## Conclusions

With the same procedure that was used for simple elastic solids, we deduce the constitutive laws for the three-phase-lag dipolar thermoelastic solids. To prove the consistency of the mixed problem formulated in this context, we formulate and argue two qualitative results, namely uniqueness and reciprocal results. These are obtained without resorting to known processes (for example, without using Laplace’s transform). Instead of these procedures, in proving the uniqueness result, for instance, the dissipative inequality is used. Also, for the general case of an anisotropic and inhomogeneous material, we prove a variational principle which generalizes the known convolutional variational principle of Gurtin in order to cover the three-phase-lag dipolar thermoelasticity theory.
